# Tracking fungal pathogens on cancer: Oncomicrobes or opportunistic bystanders?

**DOI:** 10.1371/journal.ppat.1014179

**Published:** 2026-05-07

**Authors:** Rosana Alves, Wouter Van Genechten, Patrick Van Dijck

**Affiliations:** 1 Centro de Biologia Molecular e Ambiental (CBMA), Instituto de Ciências e Inovação para a BioSustentabilidade (IB-S), Escola de Ciências, Universidade do Minho, Campus de Gualtar, Braga, Portugal; 2 Laboratory of Molecular Cell Biology, Institute of Botany and Microbiology, KU Leuven, Kasteelpark Arenberg, Leuven, Belgium; University of Maryland, Baltimore, UNITED STATES OF AMERICA

## 1. The unique microbiome of tumors

The primary habitat of the human microbiota is the gastrointestinal tract, but microbial populations exist throughout the human body including in the skin, oral, respiratory, and genital tracts. Tumors, once believed to be sterile environments, harbor unique communities of microorganisms that can influence cancer initiation, progression, and therapeutic response [1]. The ecosystems created by these communities, the microbiomes, have been associated with both cancer-promoting and protective phenotypes, consequently facilitating or protecting against tumor development [2,3]. While the tumor-associated bacteriome and virome have been extensively characterized [1–5], considerably little attention has been given to the mycobiome. Though, emerging evidence suggests that several fungal species exert complementary effects on tumor biology. Understanding these complex microbial interactions within the tumor microenvironment is opening new avenues for diagnostic and therapeutic interventions.

## 2. Fungi detected in tumors

**Fig 1:**
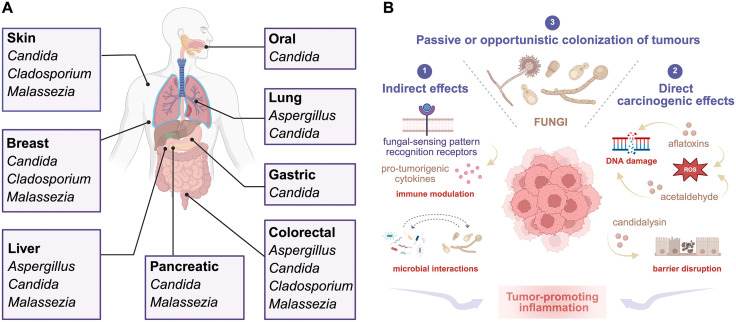
Influence of the human mycobiota on cancer. **A.** Major fungal genera detected across different cancer types. **B.** Proposed mechanisms by which fungal pathogens influence tumorigenesis. Created in BioRender. Alves, R. (2026) https://BioRender.com/cbpcf5t.

Despite their low abundance relative to cancer cells, advances in microbial cultivation, next-generation sequencing, and bioinformatic technologies are enabling a complete characterization of the tumor mycobiome (Fig 1). Recent studies have recognized intratumoral fungi as metabolically active, with specific species associated with disease state and, in some cases, patient survival [6,7]. Across studies, *Candida*, *Aspergillus*, *Malassezia*, and *Cladosporium* are among the most frequently detected fungal genera.

*Candida* species, particularly *C. albicans* and *C. tropicalis*, have been reported in oral [6,8–11], pancreatic [12–14], gastric [6], colorectal [6,15–21], hepatic [22,23], lung [7,24], and skin cancers [25]. Although the strength and consistency of these associations vary across studies, many are supported either by significant enrichment in tumor tissues compared with healthy controls [9,12,13,15,19,22,23,25] or by functional validation in mouse models [8,17,18,20,21,24,26]. Expansion of *Malassezia* has been described in breast [6,27], skin [25], liver [22], pancreatic [7,28,29] and colorectal [16,30] cancers, with some studies suggesting tissue-specific colonization patterns [16,29]. *Aspergillus* species have been identified in lung adenocarcinoma [7,31] and colorectal tumors [16,32,33], while *Cladosporium* species have been documented in melanoma [7], breast [7,34], and colorectal cancers [16]. However, evidence for their functional contribution remains more limited and context-dependent.

By contrast, potentially beneficial or commensal fungi, including *Saccharomyces, Cyberlindnera*, *Schizosaccharomyces, Pichia,* and *Debaryomyces* have also been detected across multiple datasets [6,7,25], in some cases correlating with more favorable clinical outcomes. However, whether these associations reflect functional contributions or indirect markers of environmental or dietary exposure remain unclear.

Nonetheless, interpretation of these findings remains challenging [35]. While several studies have incorporated matched adjacent tissues or healthy controls, supporting the notion of tumor-associated fungal signatures, many fungal signals are detected near the limits of sequencing sensitivity. This raises the possibility that some taxa represent laboratory contaminants or transient passengers rather than true intratumoral residents. Moreover, mislabeled sequences in reference databases can generate additional artificial signals. Together, these limitations highlight the need for rigorous experimental design, appropriate controls, and validation strategies in tumor mycobiome studies.

## 3. Oncogenic potential or opportunistic colonization: Moving beyond correlative studies

Whether specific fungal species consistently survive, replicate, and functionally influence tumor physiology is now an active area of investigation. Mechanistic studies are beginning to provide insights into the causal roles of individual fungi in cancer initiation and progression. To date, numerous and varied mechanisms have been proposed, although many remain inconclusive. While most fungal contributions to carcinogenesis appear to be mediated through chronic inflammation, some species may also exert direct genotoxic effects (Fig 1B).

### 3.1 Indirect effects via immune modulation and microbial interactions

In progressive and invasive models of pancreatic cancer, depletion of the mycobiome is protective against tumor growth, whereas repopulation with *Malassezia* species is sufficient to accelerate tumorigenesis [29]. Two independent studies linked these phenotypes to fungal-sensing pattern recognition receptors and the activation of downstream signaling pathways. Specifically, activation of a Dectin-1-dependent pathway by fungal-derived components triggered the secretion of the pro-tumorigenic cytokine IL-33 [28]. Ligation of mannose-binding lectin to fungal cell wall glycans was also shown to activate the complement cascade, which was required for oncogenic progression [29]. Indeed, complement activation has previously been associated with cancer progression, and chronic inflammation is a well-recognized driver of tumorigenesis [1]. Dectin-3, another fungal recognition receptor, has also been reported to mediate the pro-tumorigenic effect of *C. tropicalis*, in a murine model of colorectal cancer, through the activation of the NLRP3 inflammasome [36]. This multiprotein complex, present in myeloid-derived suppressor cells, regulates inflammatory signaling cascades by activating caspase-1 and the pro-inflammatory cytokine IL-1β, leading to chronic inflammation. Collectively, these findings highlight that fungal sensing by innate immune receptors can reshape the tumor microenvironment toward a pro-tumorigenic state by promoting chronic inflammation, immunosuppression, and cytokine signaling. This concept is further supported by human immunodeficiency conditions, such as Autoimmune PolyEndocrinopathy-Candidiasis-Ectodermal Dystrophy (APECED) and STAT1 gain-of-function mutations. Both conditions are characterized by susceptibility to chronic mucosal fungal infections [11,37,38] and an increased risk of squamous cell carcinoma [10,11], underscoring the importance of the immune-fungal-epithelial axis in carcinogenesis.

In addition, fungal effects on tumor biology are likely influenced by interactions with other microbial communities within the tumor microenvironment. Emerging evidence suggests that fungi can act synergistically with bacterial pathogens to promote tumor progression. For example, cooperative interactions between *C. albicans* and *Fusobacterium nucleatum* have been implicated in colorectal cancer and predictive of poor clinical outcomes via mechanisms other than inducing colonic inflammation [21]. In particular, *C. albicans* has been shown to facilitate the translocation of *F. nucleatum* to the colonic mucosa, accelerating colorectal cancer progression in mice [21]. Conversely, antagonistic interactions may also occur, whereby fungal communities modulate or counterbalance bacteria with protective or anti-tumorigenic roles [39,40]. Together, these observations emphasize the need to consider the mycobiome within the broader context of polymicrobial ecosystems and their collective impact on host immunity and cancer progression.

### 3.2 Direct carcinogenic effects of fungi

Many tumor-associated fungi produce virulence factors and metabolites that affect epithelial integrity, immune activation, and tissue homeostasis. For example, *Candida* species secrete aspartyl proteases, phospholipases, and the pore-forming mycotoxin candidalysin [41], which disrupt epithelial barriers and promote a pro-inflammatory immune response that support tumor progression [42]. The production of acetaldehyde, a genotoxic metabolite, has also been linked to epithelial dysplasia and oral carcinogenesis [43,44]. Similar to *Candida*, *Malassezia* also secretes an arsenal of hydrolytic enzymes that degrade the extracellular matrix and destroy epithelial barriers [45]. In particular, *M. globosa* has been shown to ilicit inflammatory responses through the activation of inflammatory cytokines and inflitration of immunosuppressive cells, thereby accelerating tumor growth [27]. *Aspergillus* species are also able to produce the carcinogenic mycotoxin aflatoxin, which is a well-established driver of hepatocellular carcinoma but has also been associated with increasing gallbladder cancer risk [46,47]. The molecular mechanism of aflatoxin-induced carcinogenesis involves the activation of proto-oncogenes and mutations in the tumor suppressor genes [46].

### 3.3 Passive or opportunistic colonization of tumors

While certain fungal communities can actively contribute to tumorigenesis, the tumor microenvironment itself may also represent a particularly favorable niche for fungal colonization. Tumors are characterized by profound metabolic rewiring, including elevated glucose uptake, enhanced glycolytic flux, lactate accumulation, hypoxia, and local acidosis [1]. These conditions closely resemble ecological niches in which many fungi thrive. Several fungal species are highly adapted to low-oxygen environments, acidic pH, and fluctuating nutrient availability, and can efficiently utilize carbon sources generated by tumor metabolism. For instance, *Candida* species undergo rapid expansion under such conditions [48], and their ability to utilize lactate as a nutrient source has been proposed to confer a competitive advantage over other fungi within tumor-associated niches [24]. In addition, tumor-associated immune suppression and disrupted epithelial barriers resulting from fungal activity, further reduce constraints on fungal persistence.

Although increasing evidence supports functional roles for fungi in cancer, it remains important to consider that, in some contexts, their presence may reflect opportunistic colonization of a permissive microenvironment rather than a direct causal contribution. Current evidence linking some fungal species to cancer remains largely correlative [6,7], and definitive mechanistic proof of fungal-driven carcinogenesis is still limited. Together, these metabolic and immunological features suggest that tumors may not only be shaped by fungi but may also selectively permit or even favor fungal survival and expansion, complicating efforts to distinguish causative roles from opportunistic colonization.

## 4. The therapeutic impact of the mycobiome in cancer therapy

The detection of significant levels of fungal cells in tumors compared with healthy controls has positioned fungi as promising diagnostic and prognostic biomarkers [29]. Beyond their role in carcinogenesis, alterations in fungal composition and diversity have also been reported to influence responses to chemotherapy, radiotherapy, and immunotherapy [25,49–51], suggesting that fungal communities may modulate therapeutic efficacy.

*C. tropicalis* was the first fungal pathogen shown to promote chemoresistance to oxaliplatin through lactate-induced metabolic rewiring in colon cancer [20], a mechanism that has also been recently described for tumor-associated bacteria [52]. Mechanistically, *C. tropicalis* increases tumor cell glycolysis, leading to elevated lactate production [20]. In turn, lactate downregulates the mismatch repair system in tumor cells via the GPR81-cAMP-PKA-CREB pathway, resulting in the accumulation of genetic mutations that favor resistance to oxaliplatin [20]. Elevated intratumoral lactate levels have been consistently correlated with tumor biology aggressiveness and poor survival across cancer types, contributing to both chemotherapy and radiotherapy resistance [52]. Notably, *Candida* overgrowth has also been associated with an increased abundance of lactate-producing bacteria in cancer patients [24]. Together, these observations suggest the existence of a lactate-driven positive feedback loop in which tumor cells and lactate-producing bacteria generate a metabolic niche that selectively favors *Candida* persistence and expansion, while *Candida*-induced metabolic rewiring further amplifies lactate availability. Such reciprocal interactions may reinforce ecological selection and enable efficient fungal colonization within the tumor microenvironment.

Moreover, depletion of intestinal fungi has been reported to enhance radiotherapy responsiveness in mouse models of breast and skin cancer through a Dectin-1-dependent mechanism that alters antitumor immunity [51]. In particular, mice colonized with *C. albicans* exhibited accelerated tumor regrowth following radiotherapy, accompanied by an increased ratio of immunosuppressive cells, increased cell proliferation, and reduced cell death [51]. These phenotypes were fully reversed in fluconazole-treated, fungal-free, or Dectin-1-deficient mice, supporting the notion that sensing of fungal-derived products by Dectin-1 promotes an immunosuppressive tumor microenvironment and impairs radiotherapy efficacy [51]. Of note, some effects previously attributed to altered bacteriota on antitumor immune responses may instead reflect unappreciated reciprocal changes in the mycobiota. Further supporting an immunomodulatory role for tumor-associated fungi, increased levels of *Candida* and *Malassezia* species have been associated with immunotherapy failure [25,49,53]. Whether these associations reflect direct effects on immune pathways, indirect interactions with tumor-associated bacteria, or simply the immunological state of the host remains unclear. Modulating fungal communities through diet, probiotics, or targeted antifungals therefore represents a potential avenue for enhancing cancer therapy. Such interventions will require rigorous mechanistic and clinical studies to establish whether manipulating fungal populations in humans can safely alter tumor progression or treatment response.

## 5. Conclusions and future directions

The study of the tumor-associated mycobiota presents significant opportunities to uncover new determinants of cancer initiation, progression, and therapeutic response. Realizing this potential will require deliberate efforts to enlarge epidemiological studies and standardize metagenomic workflows, including sample collection, DNA extraction, sequencing strategies, and computational pipelines [54]. This will improve reproducibility and maximize comparisons across studies. Advancing from correlative observations to mechanistic understanding will also depend on the validation of experimental models that accurately recapitulate both the human carcinogenesis process and the ecological context of inter-kingdom fungal interactions within host tissues. Preclinical models, particularly mouse models, provide valuable information and account for the majority of experimental evidence in the field. These systems offer important advantages, including functional immune responses, vascular and stromal complexity, and the ability to study whole-organism interactions in controlled settings. However, many fungal species detected in human tumors are not native to the murine microbiome, limiting the physiological relevance of colonization and immune responses in these systems. Humanized microbiota models and the use of established fungal infection systems that approximate human disease, potentially combined with carcinogenesis models, may help to mitigate these gaps. Despite their limitations, mouse models remain indispensable for dissecting causal mechanisms, although careful interpretation and validation in human systems are still required. Complementary *in vitro* approaches, such as co-culture systems pairing fungi with human tumor cells, provide rapid and tractable platforms for dissecting molecular interactions. Yet they do not capture experimentally the complexity of the tumor microenvironment or the immune system. Emerging microphysiological models, such as human organoids or organs-on-a-chip, have been successfully applied to study tumor-microbe interactions and present a promising bridge for fungal research [55–57]. By integrating human epithelial, stromal, microbial, and immune components, these systems may allow controlled mechanistic studies while preserving key features of the tumor microenvironment.

Together, these advances will help establish causality, clarify the functional repertoire of intratumoral fungi, and identify potential diagnostic, prognostic, or therapeutic applications for cancer treatment. As the field evolves, coordinated multidisciplinary efforts englobing basic research, clinical studies, and epidemiological analyses, will be essential to define how fungi shape cancer biology and how this knowledge can be applied to improve patient outcomes.
